# Early-detection and prevention effects of screening sigmoidoscopy: evidence from randomized trials revisited

**DOI:** 10.1093/jnci/djaf313

**Published:** 2025-10-30

**Authors:** Hermann Brenner, Tim Holland-Letz, Annette Kopp-Schneider, Thomas Heisser, Michael Hoffmeister

**Affiliations:** Cancer Prevention Graduate School, German Cancer Research Center (DKFZ), Heidelberg, Germany; Division of Biostatistics, German Cancer Research Center (DKFZ), Heidelberg, Germany; Division of Biostatistics, German Cancer Research Center (DKFZ), Heidelberg, Germany; Cancer Prevention Graduate School, German Cancer Research Center (DKFZ), Heidelberg, Germany; Division of Clinical Epidemiology of Early Cancer Detection, German Cancer Research Center (DKFZ), Heidelberg, Germany

## Abstract

**Background:**

Large-scale randomized controlled trials (RCTs) have established compelling evidence that screening by flexible sigmoidoscopy reduces colorectal cancer (CRC) incidence. Reported incidence results include cancers that were already prevalent and yet undiagnosed, but no longer preventable at screening. We aimed to derive, disentangle, and fully disclose early-detection and long-term prevention effects of screening sigmoidoscopy from published trial results.

**Methods:**

We used data from 3 large-scale RCTs from the United Kingdom (UKFSST), Italy (SCORE), and the United States (PLCO), which included a total number of 359 198 participants. For each trial and each length of follow-up, we derived the numbers and proportions of CRC cases that were either early detected or prevented among screening attenders.

**Results:**

In the UKFSST, which reported the longest follow-up data, screening sigmoidoscopy prevented 64% (95% CI = 59% to 69%) of incident distal CRC that would have been expected in the absence of screening during a median of 21.3 years. Within follow-up periods between 10 and 12 years, the proportions of distal CRC cases that were either early detected or prevented among screening users ranged between 67% (95% CI = 61% to 72%) in the PLCO and 80% (95% CI = 68% to 89%) in the SCORE trial, with approximately equal shares of early-detected and prevented cases in the SCORE and the PLCO trials, and a higher share of prevented cases in the UKFSST.

**Conclusions:**

A single screening sigmoidoscopy prevents 2 out of 3 incident cancers in the distal colon and rectum over a period of more than 20 years, on top of early-detecting prevalent cases at screening.

## Introduction

Colorectal cancer (CRC) is the third most common cancer and the second most common cause of death due to cancer globally.^1^ The majority of colorectal cancers are located in the distal colon and rectum. Four large-scale randomized controlled trials (RCTs) have established compelling evidence that screening by flexible sigmoidoscopy, which visualizes the distal colon and rectum, can substantially reduce CRC incidence and mortality, in particular incidence and mortality from distal CRC.^2-11^

In these trials, following standard RCT methodology, effects on CRC incidence and mortality have primarily been reported as intention-to-screen estimates, comparing CRC incidence and mortality between participants who were invited for screening and those who were not invited. In addition, 3 of the trials also reported adjusted per-protocol estimates to quantify the effects that would have been expected if all invited participants had followed the invitation. However, for cancer screening studies, observed “incidence” is a complex outcome that may include both cases that were already prevalent but yet undiagnosed at the time of screening. Such cases would therefore no longer be preventable by screening, but they could be early detected, which may enhance chances of cure, another desirable effect of screening.^12^^,^^13^ Because the standard intention-to-screen and per-protocol analyses of the RCTs do not differentiate between early detection and prevention effects of screening, we recently developed an alternative analytical approach and applied it to disentangle both components of screening effects for screening colonoscopy based on the NordICC trial, the first RCT on long-term effects of screening colonoscopy.^14^^,^^15^ We thereby demonstrated much stronger effects of screening colonoscopy than those reflected in the reported intention-to-screen and per-protocol estimates of incidence reduction.

In this article, we apply the alternative methodology that preserves the unique strengths of the RCT design to published results from 3 of the large screening sigmoidoscopy RCTs, which reported results in sufficient detail to do so. We thereby aim to disentangle and fully disclose the effects of screening sigmoidoscopy on both CRC early detection and prevention.

## Methods

### Database

Details on the design and results of the sigmoidoscopy trials have been reported elsewhere and are summarized in Table 1. One of the 4 sigmoidoscopy trials, the Norwegian Colorectal Cancer Prevention (NORCCAP) Screening Study,^9^^,^^10^ was not included in our analyses, as it had made an additional offer of a fecal occult blood test to a random sample of 50% of participants of the intervention group, had used different randomization ratios and different lengths of follow-up for various age groups, and did not report overall and site-specific results in sufficient detail. The other 3 trials, conducted in the United Kingdom, Italy, and the United States, included the United Kingdom Flexible Sigmoidoscopy Screening Trial (UKFSST),^2-4^ the Screening for COlon REctum (SCORE) trial,^5^^,^^6^ and the Prostate, Lung, Colorectal, and Ovarian (PLCO) Cancer Screening Trial.^7^^,^^8^ In these trials, men and women at various age ranges between 50 and 74 years were recruited during various periods between 1994 and 2001 and randomized to the offer of screening sigmoidoscopy (single screening sigmoidoscopy in UKFSST and SCORE, up to 2 sigmoidoscopies 3 or 5 years apart in PLCO). Sample sizes ranged from 34 272 in SCORE to 170 026 in the UKFSST. Screening uptake rates in the intervention group ranged from 57.8% in SCORE to 86.6% in PLCO.

**Table 1.** Key design features and reported main results on CRC incidence reduction of the randomized trials included in this analysis.

Feature/Metric	Trial					
	UKFSST^2-4^			SCORE^5^^,^^6^		PLCO^7^
Country	United Kingdom			Italy		United States
Screening offer	Single sigmoidoscopy			Single sigmoidoscopy		2 screening sigmoidoscopies 3 or 5 years apart
Age at recruitment	55-64			55-64		55-74
Years of recruitment	1994-1999			1995-1999		1993-2001
Recruited, *N*	170 026			34 272		154 900
Control group, participants, *n*	112 927			17 136		77 455
Intervention group, participants, *n*	57 099			17 136		77 445
Screening attenders, *n* (%)	40 621 (71.1%)			9911 (57.8%)		67 071 (86.6%)^a^
Screening attenders, early-detected cases, *n*	140			54		244^b^
Median follow-up	11.2 years	17.1 years	21.3 years	10.5 years	15.4 years	11.9 years
Control group, observed cases, *N*	1818	3253	4201	306	468	1287
Intervention group, observed cases, *n*	706	1230	1631	251	382	1012
Screening attenders, observed cases, *n*	445	776	1052	126	184	851
Incidence reduction (95% CI), %						
All CRC, intention-to-screen analysis	23 (16 to 30)	26 (20 to 30)	24 (19 to 28)	18 (4 to 31)	19 (7 to 29)	21 (15 to 28)
All CRC, per-protocol analysis	33 (24 to 40)	35 (29 to 41)	32 (27 to 37)	31 (14 to 44)	33 (19 to 44)	n.r.
Distal CRC, intention-to-screen analysis	36 (28 to 43)	41 (36 to 46)	41 (36 to 46)	24 (6 to 38)	30 (16 to 41)	29 (20 to 36)
Distal CRC, per-protocol analysis	50 (41 to 58)	56 (50 to 62)	56 (50 to 61)	40 (20 to 54)	50 (37 to 61)	n.r.

Abbreviations: CI = confidence interval; CRC = colorectal cancer; FOBT = fecal occult blood test; PLCO = Prostate, Lung, Colorectal, and Ovarian; SCORE = Screening for COlon REctum; UKFSST = United Kingdom Flexible Sigmoidoscopy Screening Trial.

a Uptake of at least 1 screening sigmoidoscopy.

b Detected at first or second screening sigmoidoscopy.

Primary endpoints in all trials included CRC incidence and mortality. In our analysis, we focus on CRC incidence results, which were reported after median follow-up times between 10.5 and 21.3 years, with the UKFSST reporting the longest follow-up. Even though results of PLCO were also reported after a longer follow-up period than the one listed in Table 1,^8^ we did not include these results as the later PLCO report had not provided data on CRC cases according to screening attendance which are required for our analyses. Overall, the 3 trials reported quite consistent results. For CRC at any site, incidence reductions between 18% and 26% were reported in intention-to-screen analyses. In per-protocol analyses, estimated incidence reduction if all participants accepted the screening ranged from 31% to 35%. For distal CRC, stronger risk reductions were reported, ranging from 24% to 41% in intention-to-screen analyses and from 40% to 56% in per-protocol analyses.

### Statistical analysis

Based on the published trial data and results, we derived the following metrics to unravel and fully disclose the effects of early detection and prevention among screening attenders:

- Expected numbers of CRC cases in the absence of screening.
- Proportions of early-detected and prevented CRC among these expected numbers.

It is worth noting that the term early-detected CRC cases, as used in our article, includes all screen-detected cases. Even though screen-detected cases also include a small proportion of CRC cases detected at advanced stages, all of the screen-detected cases were still detected earlier by screening than they would have been detected without screening.

**Figure 1.**
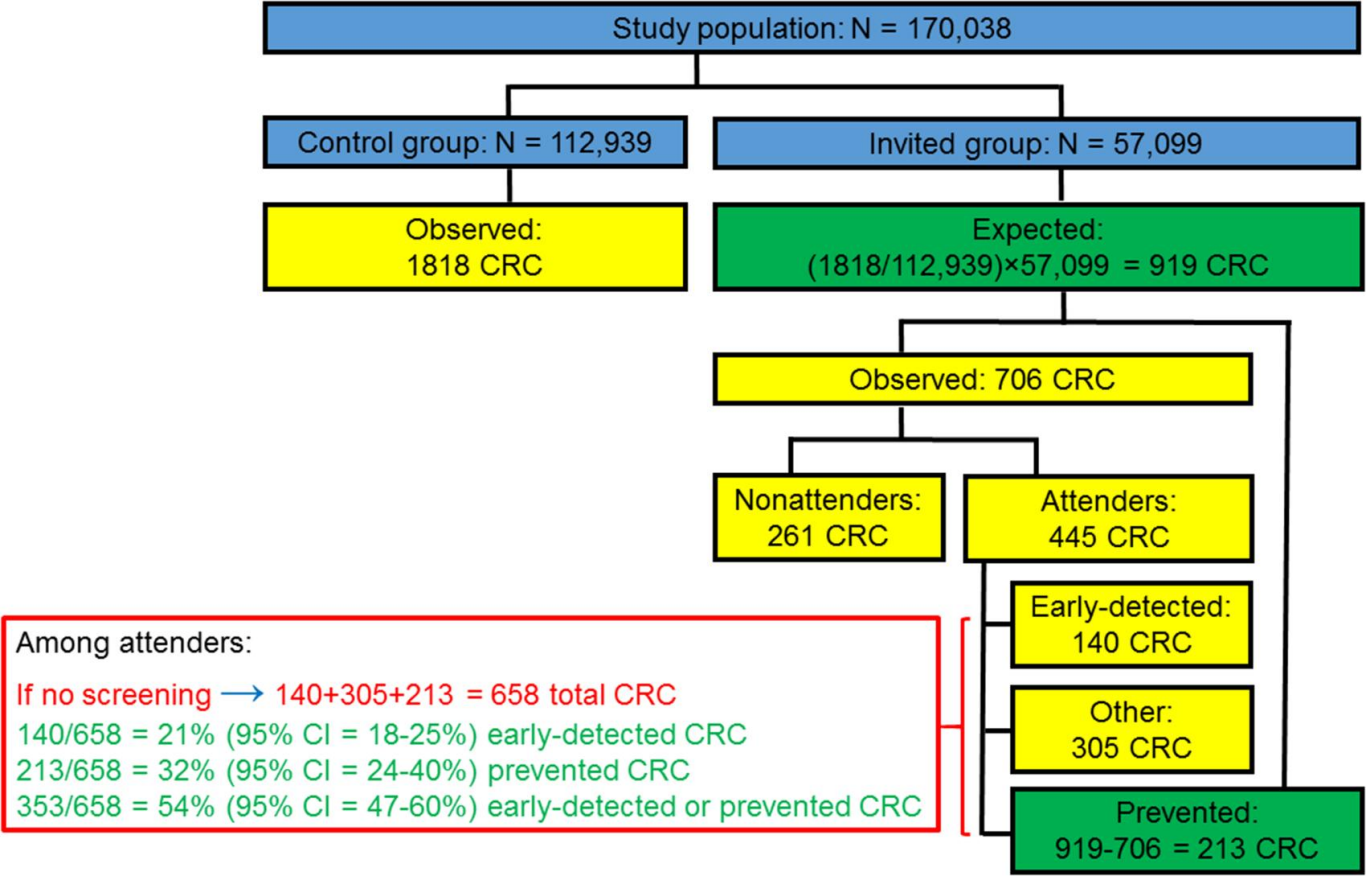
Derivation of the proportions of early-detected and prevented total colorectal cancer (CRC) cases among screening attenders after 11.2 years of follow-up in the United Kingdom Flexible Sigmoidoscopy Screening Trial.^2^

Table 2 and Figure 1 illustrate our derivation for CRC at any site based on data reported from the UKFSST after a median follow-up of 11.2 years^2^: As previously explained in detail for analogous analyses regarding the preventive effects of screening colonoscopy,^15^ our analysis is based on just 2 basic, highly plausible assumptions:

**Table 2.** Exemplary calculation of the proportions of early-detected and prevented CRC cases among screening attenders based on data reported from the UKFSST after a median follow-up of 11.2 years.^2^

Group, metric	Abbreviation	Reported no.	Calculation	Result
Control group				
Participants	M1	112 939		
Observed CRC cases	M2	1818		
Intervention group				
Participants	M3	57 099		
Observed CRC cases	M4	706		
Expected CRC cases^a^	M5		M3 × (M2/M1)	919
Prevented CRC cases	M6		M5 − M4	213
Intervention attenders				
Observed CRC cases	M7	445		
Early-detected CRC cases	M8	140		
Expected CRC cases^a^	M9		M7 + M6	658
% early-detected cases	M10		100 × (M8/M9)	21%
% prevented cases	M11		100 × (M6/M9)	32%
% early-detected or prevented cases	M12		M10 + M11	54%^b^

Abbreviations: CRC = colorectal cancer; UKFSST = United Kingdom Flexible Sigmoidoscopy Screening Trial.

a Expected in the absence of the screening offer.

b Apparent deviation from the sum of M10 and M11 is due to rounding.

- Randomization in these large trials ensured equal CRC risk in the invited group and the control group in the absence of the screening offer (“standard RCT assumption”).
- Screening colonoscopy could prevent CRC only among those who attended it, that is, prevented cases were exclusively prevented among screening attenders. It is worth noting that this assumption holds regardless of potential selective use of screening by people at increased or decreased CRC risk in the invited group, a common concern in standard per-protocol analyses.

In a first step, we calculated the expected number of CRC cases in the invited group (*n* = 57 099) by assuming that, given the randomization, the proportion of participants who were diagnosed with CRC should have been the same as in the control group (*n* = 112 939):


Expected number of CRC cases in the invited group=(1818/112 939)×57 099=919.

In a second step, we calculated the number of prevented CRC cases in the invited group as the difference between the expected number in the absence of the screening offer (*n* = 919) and the observed number (*n* = 706):


Prevented number of CRC cases=919−706=213.

CRC cases could have been prevented by screening sigmoidoscopy only among those who actually used it. In a third step, the expected number of CRC cases among screening attenders in the absence of screening was therefore calculated as the sum of observed CRC cases in this group (*n* = 445) and the prevented cases (*n* = 213):


Expected number of CRC cases among screening attenders=445+213=658.

Finally, we quantified the proportions of early-detected CRC cases (*n* = 140), prevented CRC cases (*n* = 213), and either early-detected or prevented cases (*n* = 140 + 213 = 353) as the proportion of the number of expected CRC cases among the screening attenders, that is, as 140/658 (21%), 213/658 (32%), and 353/658 (54%), respectively. It is worth noting that the derivation of the proportion of prevented cases among screening attenders corresponds, apart from using count data rather than person-time data, to the derivation of per-protocol estimates of prevention effects, derived by the method proposed by Cuzick et al^16^ in the original trial publication.

We derived 95% confidence intervals for the estimated proportions of prevented and early-detected cases as the 2.5th and 97.5th percentiles of 1 million runs of Monte Carlo simulations. For each simulation run, the case numbers were drawn randomly as follows, using the original cohort sizes as *n* and using the attendance and CRC case proportions derived from the observed numbers as expected values: (1) The cohort size of the invited group was randomly split into attenders and nonattenders using a binomially distributed random variable. (2) A random number of overall CRC cases was drawn independently for both the control group and the group of invited nonattenders from a binomial distribution. (3) Both prevalent and incident case numbers were drawn randomly and simultaneously for the group of attenders from a multinomial distribution. (4) All relevant CRC proportions were then calculated for this simulation run from the obtained numbers.

**Figure 2.**
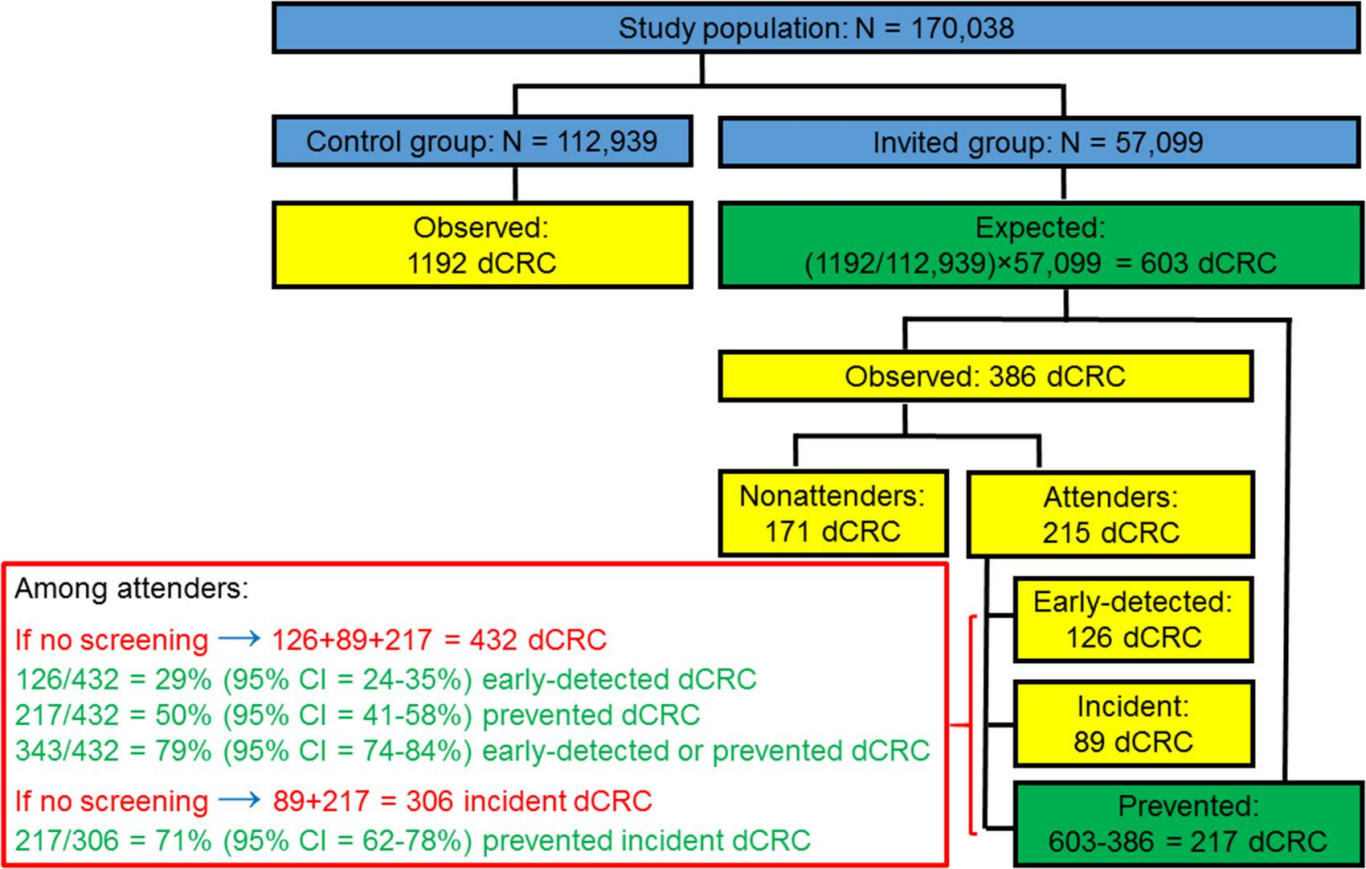
Derivation of the proportions of early-detected and prevented distal colorectal cancer (dCRC) cases among screening attenders after 11.2 years of follow-up in the United Kingdom Flexible Sigmoidoscopy Screening Trial.^2^

As illustrated in **Table S1** and Figure 2, analogous calculations can be made for distal CRC. In addition, for distal CRC, a clear distinction can be made among screening attenders between observed *prevalent* cases (which were detected at screening sigmoidoscopy, *n* = 126) and observed *incident* cases (*n* = 89). This way, the number of expected *incident* cases in the absence of screening could additionally be calculated as the sum of the observed incident cases (*n* = 89) and the prevented cases (*n* = 217), that is, as *n* = 89 + 217 = 306, and the proportion of prevented incident cases could be calculated as 217/306 = 71%. Note that the distinction between prevalent and incident cases cannot be made with certainty for CRC at any site (Figure 1), because the observed “other” (not early-detected) cases among screening attenders could include prevalent proximal CRC cases that were already present at baseline but had remained undetected at that time.

We carried out analogous calculations based on UKFSST results reported after median follow-ups of 17.1 and 21.3 years, and based on reported data after various lengths of follow-up from the SCORE and PLCO trials. Data used for these calculations, which we extracted from the trial publications, are shown in **Table S2**. For the PLCO trial, early-detected CRC cases included those detected at either the first or the second screening sigmoidoscopy.

## Results

As shown in Table 3, the proportions of early-detected CRC at any site among CRCs expected in screening attenders within follow-up periods between 10 and 12 years was rather consistently estimated as between 20% and 30%. For obvious reasons, this proportion decreased with additional CRC diagnoses expected during longer follow-up periods. By contrast, the estimated proportions of prevented CRC remained rather constant across different trials and different lengths of follow-up and ranged from 24% after a median follow-up of 11.9 years in the PLCO trial to 35% after a median follow-up of 17.1 years in the UKFSST. Overall, approximately half of CRC cases located at any site that were expected among screening attenders in the absence of screening were either early detected or prevented, with estimates ranging from 41% (95% CI = 36% to 46%) for the UKFSST results after a median follow-up of 21.3 years to 60% (95% CI = 45% to 71%) for the SCORE results after a median follow-up of 10.5 years.

**Table 3.** Proportions of early-detected and prevented CRC cases among screening attenders.

Metric		Study, median length of follow-up					
		UKFSST 11.2 years^2^	UKFSST 17.1 years^3^	UKFSST 21.3 years^4^	SCORE 10.5 years^5^	SCORE 15.4 years^6^	PLCO 11.9 years^7^
Proportions of all CRC, % (95% CI)	Early detected	21 (18 to 25)	12 (10 to 14)	9 (8 to 11)	30 (21 to 42)	20 (14 to 27)	22 (19 to 25)
	Prevented^a^	32 (24 to 40)	35 (29 to 41)	32 (26 to 37)	30 (6 to 47)	32 (13 to 46)	24 (17 to 31)
	Early detected or prevented	54 (47 to 60)	47 (41 to 52)	41 (36 to 46)	60 (45 to 71)	52 (38 to 62)	46 (40 to 51)
Proportions of distal CRC, % (95% CI)	Early detected	29 (24 to 35)	17 (14 to 20)	14 (11 to 17)	41 (27 to 62)	27 (19 to 39)	35 (29 to 41)
	Prevented^a^	50 (41 to 58)	56 (50 to 61)	55 (50 to 60)	39 (11 to 57)	50 (31 to 63)	33 (23 to 41)
	Early detected or prevented	79 (74 to 84)	73 (69 to 77)	69 (65 to 73)	80 (68 to 89)	77 (66 to 85)	67 (61 to 72)
	Prevented among incident cases	71 (62 to 78)	67 (62 to 73)	64 (59 to 69)	67 (27 to 82)	68 (49 to 80)	50 (38 to 59)

Abbreviations: CI = confidence interval; CRC = colorectal cancer; PLCO = Prostate, Lung, Colorectal, and Ovarian; SCORE = Screening for COlon REctum; UKFSST = United Kingdom Flexible Sigmoidoscopy Screening Trial.

a These estimates, which are based on count data, are very close to the per-protocol estimates reported in the original trial publications, which had been derived by the method of Cuzick et al^16^ based on person-time data.

For distal CRC, much higher proportions of both early-detected and prevented cases were estimated. The proportions of cases that were either early detected or prevented were estimated between 67% (95% CI = 61% to 72%) in the PLCO trial and 80% (95% CI = 68% to 89%) after median follow-up of 10.5 years in the SCORE trial, with approximately equal shares of early-detected and prevented cases in the SCORE and the PLCO trials, and a higher share of prevented cases in the UKFSST. The proportions of prevented incident distal cancers were estimated between 50% (95% CI 38% to 59%) in the PLCO trial and 71% (95% CI = 62% to 78%) after a median follow-up time of 11.9 years in the UKFSST. In the UKFSST, the proportion of prevented incident distal CRC was still as high as 64% (95% CI = 59% to 69%) even after a median follow-up of 21.3 years.

## Discussion

In this re-analysis of published data from the large screening sigmoidoscopy trials, we demonstrate that use of a single screening sigmoidoscopy enabled early detection or prevention of approximately 4 out of 5 distal CRC that would otherwise have been expected within 10-12 years among people who had a first screening endoscopy between 50 and 74 years of age. Furthermore, we demonstrate that screening sigmoidoscopy prevented approximately 2 out of 3 incident distal CRC cases during follow-up periods of up to and beyond 20 years. Given the previously demonstrated essential lack of effects on proximal CRC, it is not surprising that effects for overall CRC were somewhat less pronounced. However, as the majority of CRC are located in the distal colon or rectum, proportions of early-detected or prevented cases among all CRC were still substantial, with approximately 1 out of 2 expected cases having been early detected or prevented among screening attenders even in the very long run. These results underline the strong and long-lasting preventive effects of even a single screening sigmoidoscopy.

In previous work,^17^ we had derived proportions of prevented CRC during 11.2 and 17.1 years of follow-up for the UKFSST,^2^^,^^3^ and during 10.5 and 15.4 years of follow-up for the similarly designed (but much smaller) Italian SCORE trial.^5^^,^^6^ The current analysis expands previous findings by including the most recently published 21-year follow-up data of the UKFSST,^9^ by additionally including data from the large PLCO trial, and by deriving additional metrics to unravel and more comprehensively describe the effects of screening sigmoidoscopy on early detection of prevalent cases and prevention of incident CRC cases.

We have recently derived, for the first time, similar metrics for the NordICC trial,^15^ so far the only RCT on long-term effects of screening colonoscopy,^14^ which visualizes the entire colon and rectum. The estimated proportions of close to 80% of either early-detected or prevented distal CRC within 10 to 12 years of follow-up derived in the current analysis are even slightly stronger than the corresponding estimate of 74% of early-detected or prevented cancers in the entire colon and rectum in the NordICC trial after a median follow-up of 10 years. Furthermore, the estimated proportions of prevented incident distal CRC by screening sigmoidoscopy in the current analysis, which ranged from 64% to 72% across trials and various follow-up times, were even substantially higher than the corresponding estimate for prevented all-site CRC in NordICC (57%). Apart from specific limitations of the NordICC trial, such as shorter and likely incomplete follow-up due to delayed cancer registration and differential postrandomization exclusions,^18^^,^^19^ these patterns may reflect even stronger preventive effects of endoscopic screening in the distal colon and rectum than in the proximal colon, in agreement with observational epidemiological studies.^20-22^

It is worth noting that the estimates of the prevented proportions among all CRCs (ie, among both early-detected and incident CRC combined), which pertain to people actually using screening sigmoidoscopy and which have previously been proposed as per-protocol estimates of screening effects by Cuzick et al,^16^ are not affected by potential bias by selective use of the screening offer, a common concern for other conventional per-protocol analyses. In fact, all of our estimates are based on the number and proportion of prevented CRC cases which was derived by standard intention-to-screen comparison of the invited group and the control group. Apart from the standard RCT assumption (equal risks in the invited group and the control group), the only additional assumption needed for our analyses was that screening sigmoidoscopy could exert its preventive effects only among those who actually used it, which is highly plausible. Although not all people offered screening sigmoidoscopy will use the screening offer, the per-protocol estimates proposed by Cuzick et al and all of the additional metrics quantifying the benefits of screening use among screening users derived in our analysis are not only less prone to potential bias but also more relevant for decisions whether to screen than other conventional per-protocol analyses. The latter quantify benefits for the hypothetical and unrealistic situation in which all people would use the screening offer.

Although reporting of effect estimates after extended follow-up periods is a major strength of the trials, in particular the UKFSST, one has to be aware of substantial contamination by diagnostic sigmoidoscopies or colonoscopies during such long-term follow-up. Such diagnostic endoscopies are expected to have similar preventive effects as screening sigmoidoscopies and thereby substantially attenuate observed screening effects.^23^^,^^24^ For example, in the European Health Interview Survey conducted in 2013-2016, more than 20% of participants aged 60-74 years from the United Kingdom reported having had a colonoscopy in the preceding 10 years.^25^ In the absence of such widespread use of diagnostic endoscopies, preventive effects of screening sigmoidoscopy would have been expected to be even substantially larger. Particularly high levels of contamination were reported in the PLCO trial conducted in the United States, where the estimated rate of endoscopic contamination in the usual-care group was as high as 25.8% for flexible sigmoidoscopy, 34.4% for colonoscopy, and 46.5% for either flexible sigmoidoscopy or colonoscopy already during the screening phase, and the rate of routine colonoscopy after the screening phase was 47.7% (95% CI = 44.7 to 50.7) in the intervention group and 48.0% (95% CI = 45.2 to 50.8) in the usual-care group.^7^ This high level of contamination may explain the somewhat lower proportions of prevented CRCs derived for this trial compared with the European trials. Also, the numbers and proportions of early-detected cancers would even be larger if cancers that were early detected at surveillance colonoscopies conducted after polypectomy, whose number was not reported, would also have been considered.

To our knowledge, this is the first analysis that systematically disentangles effects of screening sigmoidoscopy on both early-detection and prevention of both total and distal CRC in the large-scale randomized screening sigmoidoscopy trials. In the interpretation of our analyses, a number of limitations require careful discussion. First, as some of the findings had been reported in less detail from the NORCCAP trial, the proposed metrics could not be derived for this trial. Nevertheless, given the overall consistency of reported results across the 4 sigmoidoscopy trials, these metrics would not have been expected to be substantially different from those derived from the other trials. Second, the trials differed in some important design aspects, such as the age range and type of screening offers (single vs repeat screening sigmoidoscopy), and in reporting of results after different lengths of follow-up. We therefore abstained from summarizing the results by meta-analyses. However, with the exception of the proportion of early-detected cases, which decreased with increasing length of follow-up, our estimates are remarkably consistent across the different trials and different lengths of follow-up, suggesting that they are robust indicators of the effects of screening sigmoidoscopy across different settings and countries. Third, all of our analyses are based on published aggregate count data rather than individual-level person-time data that had been used in the original trial analyses. This may have led to slight inconsistencies between our and reported results. However, as demonstrated in **Table S3**, derivation of standard intention-to-screen effect estimates from published aggregate data would have yielded results that are almost identical to the reported ones, which suggests that this minor difference should be essentially negligible. Fourth, summing up the proportions of early-detected and prevented CRC cases, besides reporting the individual proportions, may be considered to yield a relatively crude summary measure of screening effects, which should be interpreted prudently and may be further refined in further research. Finally, derivation of equations for confidence intervals for our proposed effect metrics is not straightforward. However, as demonstrated in our analysis, such confidence intervals can easily be derived, with very limited computational resources, by bootstrap analyses.

In summary, our analysis provides a range of complementary metrics that comprehensively describe and evaluate the strong, long-lasting benefits of even a single screening sigmoidoscopy used between 50 and 74 years of age. Our analysis of 3 of the large screening sigmoidoscopy trials demonstrates that such a screening sigmoidoscopy prevents approximately 2 out of 3 incident distal cancers, on top of early-detecting prevalent cases at screening, and that this preventive effect prevails over more than 20 years from screening. Given the limited capacities for and use of screening colonoscopy or annual or biennial screening by fecal immunochemical test in many countries,^26^^,^^27^ these results underline the potential of screening sigmoidoscopy as an alternative or complementary, highly effective endoscopic screening approach, especially if such offer could help to increase screening use.

## Supplementary Material

djaf313_Supplementary_Data

## Data Availability

All data used for our calculations, which were drawn from previous original publications of the trial results are included in our manuscript.
