# Supplementary material for: Early-detection and prevention effects of screening sigmoidoscopy: evidence from randomized trials revisited
Source: J Natl Cancer Inst. 2025 Oct 30;118(3):422–8. doi: 10.1093/jnci/djaf313 (PMC13017212; doi:10.1093/jnci/djaf313)
Supplement: djaf313_Supplementary_Data [file djaf313_supplementary_data.zip › Supplementary Tables_rev_clean.docx]

**Supplementary Materials**

**Table S1**. Exemplary calculation of the proportions of early detected and prevented distal CRC (dCRC) cases among screening attenders based on data reported from the UKFSST after a median follow-up of 11.2 years^2^

| Group, metric | Abbreviation | Reported number | Calculation | Result |
| --- | --- | --- | --- | --- |
|  |  |  |  |  |
| Control group |  |  |  |  |
| Participants | M1 | 112,939 |  |  |
| Observed dCRC cases | M2 | 1192 |  |  |
| Intervention group |  |  |  |  |
| Participants | M3 | 57,099 |  |  |
| Observed dCRC cases | M4 | 386 |  |  |
| Expected dCRC cases^a^ | M5 |  | M3 × (M2 / M1) | 603 |
| Prevented dCRC cases | M6 |  | M5 − M4 | 217 |
| Intervention attenders |  |  |  |  |
| Observed dCRC cases | M7 | 215 |  |  |
| Early-detected dCRC cases | M8 | 126 |  |  |
| Expected dCRC cases^a^ | M9 |  | M7 + M6 | 432 |
| % early-detected dCRC cases | M10 |  | 100 × (M8 / M9) | 29% |
| % prevented dCRC cases | M11 |  | 100 × (M6 / M9) | 50% |
| % early-detected or prevented dCRC cases | M12 |  | M10 + M11 | 79% |
| Observed incident dCRC cases | M13 |  | M7 – M8 | 89 |
| Expected incident dCRC cases | M14 |  | M13 + M6 | 306 |
| % prevented incident dCRC cases | M15 |  | M6 / M14 | 71% |

CRC, colorectal cancer; dCRC, distal colorectal cancer

^a^ expected in the absence of the screening offer

**Table S2.** Participant and case numbers extracted from the trial publications that were used for our analyses

|  | Study, median length of follow-up | | | | | |
| --- | --- | --- | --- | --- | --- | --- |
|  | UKFSST  11.2 years^2^ | UKFSST  17.1 years^3^ | UKFSST 21.3 years^4^ | SCORE  10.5 years^5^ | SCORE  15.4 years^6^ | PLCO  11.9 years^7^ |
|  |  |  |  |  |  |  |
| Control group |  |  |  |  |  |  |
| Participants | 112,939 | 112,936 | 112,927 | 17,136 | 17,136 | 77,455 |
| CRC cases | 1818 | 3523 | 4201 | 306 | 468 | 1287 |
| Distal CRC cases | 1192 | 1987 | 2434 | 198 | 297 | 669 |
| Intervention group |  |  |  |  |  |  |
| Participants | 57,099 | 57,098 | 57,099 | 17,136 | 17,136 | 77,445 |
| CRC cases | 706 | 1230 | 1631 | 251 | 382 | 1012 |
| Distal CRC cases^a^ | 386 | 592 | 726 | 152 | 209 | 479 |
| Intervention attenders |  |  |  |  |  |  |
| Participants | 40,621 | 40,621 | 40,624 | 9911 | 9911 | 67,071 |
| CRC cases | 445 | 776 | 1052 | 126 | 184 | 851 |
| Distal CRC cases | 215 | 325 | 406 | 71 | 89 | 394 |
| Early-detected CRC cases | 140 | 140 | 140 | 54 | 54 | 244^a^ |
| Early-detected distal CRC cases | 126 | 126 | 126 | 48 | 48 | 202^a^ |

^a^ detected at 1^st^ or 2^nd^ screening sigmoidoscopy

**Table S3.** Comparison of reported intention-to-screen effect estimates on CRC incidence from the three screening sigmoidoscopy trials, which are based on individual-level person-time data, with corresponding effect estimates derived from reported aggregate count data

| Trial | Median  Follow-up  [years] | Relative risk (95% CI) estimates derived from … | | | |
| --- | --- | --- | --- | --- | --- |
|  |  | … individual-level person-time data | | … published aggregate count data | |
|  |  | Any CRC | Distal CRC | Any CRC | Distal CRC |
|  |  |  |  |  |  |
| UKFSST^2-4^ | 11.2 | 0.77 (0.70-0.84) | 0.64 (0.57-0.72) | 0.77 (0.70-0.84) | 0.64 (0.57-0.72) |
|  | 17.1 | 0.74 (0.70-0.80) | 0.59 (0.54-0.64) | 0.75 (0.70-0.80) | 0.59 (0.54-0.65) |
|  | 21.3 | 0.76 (0.72-0.81) | 0.59 (0.54-0.64) | 0.77 (0.73-0.81) | 0.59 (0.54-0.64) |
|  |  |  |  |  |  |
| SCORE^5,6^ | 10.5 | 0.82 (0.69-0.96) | 0.76 (0.62-0.94) | 0.82 (0.70-0.97) | 0.77 (0.62-0.95) |
|  | 15.4 | 0.81 (0.71-0.93) | 0.70 (0.59-0.84) | 0.82 (0.71-0.93) | 0.70 (0.59-0.84) |
|  |  |  |  |  |  |
| PLCO^7^ | 11.9 | 0.79 (0.72-0.85) | 0.71 (0.64-0.80) | 0.79 (0.72-0.85) | 0.72 (0.64-0.80) |

CRC, colorectal cancer
